# Field Study of the Interior Noise and Vibration of a Metro Vehicle Running on a Viaduct: A Case Study in Guangzhou

**DOI:** 10.3390/ijerph17082807

**Published:** 2020-04-19

**Authors:** Lei Yan, Zhou Chen, Yunfeng Zou, Xuhui He, Chenzhi Cai, Kehui Yu, Xiaojie Zhu

**Affiliations:** 1 School of Civil Engineering, Central South University, Changsha 410075, China; leiyan@csu.edu.cn (L.Y.); zhouchen520@csu.edu.cn (Z.C.); yunfengzou@csu.edu.cn (Y.Z.); xuhuihe@csu.edu.cn (X.H.); 164801022@csu.edu.cn (K.Y.); 2 National Engineering Laboratory for High Speed Railway Construction, Changsha 410075, China; 3 Guangzhou Metro Corporation, Ltd., Guangzhou 510000, China; zhuxiaojie@gzmtr.com

**Keywords:** sound pressure level, field measurements, spectrum analysis, interior noise and vibration of vehicle

## Abstract

The interior noise and vibration of metro vehicles have been the subject of increasing concern in recent years with the development of the urban metro systems. However, there still is a lack of experimental studies regarding the interior noise and vibration of metro vehicles. Therefore, overnight field experiments of the interior noise and vibration of a standard B-type metro train running on a viaduct were conducted on metro line 14 of Guangzhou (China). Both the A-weighted sound pressure level and linear sound pressure level were used to evaluate the interior noise signals in order to revel the underestimation of the low-frequency noise components. The results show that the interior noise concentrates in the low-to-middle frequency range. Increasing train speeds have significant effects on the sound pressure level inside the vehicle. However, two obvious frequency ranges (125–250 Hz and 400–1000 Hz) with respective corresponding center frequencies (160 Hz and 800 Hz) of the interior noise are nearly independent of train speed. The spectrum analysis of the vehicle body vibration shows that the frequency peak of the floor corresponds to the first frequency peak of the interior noise spectrum. There are two frequency peaks around 40 Hz and 160 Hz of the sidewall’s acceleration level. The frequency peaks of the acceleration level are also independent of the train speeds. It hopes that the field measurements in this paper can provide a data set for researchers for further investigations and can contribute to the countermeasures for reducing interior noise and vibration of a metro vehicle.

## 1. Introduction

In recent decades, the urban metro system has seen rapid development in eastern countries due to its fast speed, high efficiency, comfort, and environmental benefits, especially in China. According to recent statistics, the total length of China’s operating metro lines has increased from 112 km among three cities in 2000 to about 6000 km among 41 cities in 2019. The elevated metro has become an alternative rather than the general underground metro type due to its low cost and short construction period, especially in a metropolis with a well-developed metro network, such as Beijing, Shanghai, and Guangzhou. The total length of operated elevated metros in China accounts for nearly a quarter of the whole 6000 km [1]. Although many benefits come from urban rail transit, the rail transit system has been considered the second greatest noise source affecting human modern lifestyles [2,3,4], after road traffic [5,6,7], but before airports [8,9], industries and wind turbines [10,11], and port activities [12,13]. Moreover, there are growing complaints about the noise inside the trains, as more passengers and metro staff will be exposed to interior train noise for a longer time with the expansion of the metro system [14,15]. According to statements released by the World Health Organization (WHO), noise exposure to 85 dB A-weighted over 45 min will lead to noise-induced hearing loss (NIHL) [16]. Meanwhile, recent investigations reveal that the accumulation of short-term noise exposure can also cause NIHL [17]. Furthermore, sleep disorders with awakenings, learning impairment, hypertension, ischemic heart disease and especially annoyance are the most common negative health effect related to prolonged exposure [18,19,20,21].

The interior noise inside a railway vehicle is composed of air-borne and structure-borne sound generated by exterior sources mainly including wheel–rail rolling effects, excitation phenomena due to the sleeper-passing frequency, and aerodynamic effects [22]. Many achievements concerning the interior noise problem of trains have been made theoretically and numerically. Eade and Hardy [23] investigated the transmission mechanisms of noise generated by various sources into a vehicle train through both the air-borne and structure-borne path. The spectrum results of interior noise show that low-frequency noise accounted for the largest proportion. Forssén et al. [24] proposed a statistical energy analysis model to predict the interior sound field of a railway vehicle. The prediction results were validated by a ray tracing method and scale model measurements. Zheng et al. [25] established a full-spectrum prediction method of the interior noise of high-speed train by considering both the air-borne and structural-borne noise. Dai et al. [26] presented a prediction method by applying statistical vibration and acoustic energy flow to obtain the full-spectrum interior noise of high-speed railway vehicles. Shi [27] employed a dynamic train-track interaction model, a finite element model, and an acoustic boundary element model to predict the interior noise of a high-speed vehicle at a speed of 200 km/h.

In addition to the aforementioned theoretical and numerical investigations, many field measurements have also been conducted. Han et al. [28] investigated the effects of rail corrugation on the interior noise and vibration of a metro vehicle based on the measurements. Li et al. [29] conducted field measurements to examine the interior noise and vibration of a railway vehicle at different speeds with respect to two different rail fastener stiffnesses. Fan et al. [30] analyzed the major interior noise sources and their corresponding transmission path into a high-speed vehicle through acoustic-vibration measurements as the train travels. Thompson [22] concluded that the low-frequency noise in a trail vehicle were the results of the structure-borne noise, while the air-borne noise contributed to the high-frequency noise of the train’s interior noise according to measured results in British coaches. Conventionally, the A-weighted sound pressure level (SPL) has been widely used to evaluate the acoustical environment inside the train, and it has already been incorporated into some standards [31,32]. However, acoustic comfort inside the train cannot be achieved even when the A-weighted SPL inside the train meets the requirements of these standards due to the underestimated impacts of the low-frequency components of interior noise on people in the A-weighted SPL evaluation [33,34]. Soeta et al. [35] pointed out that the improvement of the acoustic environment in a train’s carriages only through the reduction of the A-weighted SPL inside the carriage was impossible. The subjective felling should be considered in the evaluation of acceptable interior noise inside a train vehicle [22,36].

As there remains great concern regarding the acoustic environment of a metro vehicle, the lack of experimental studies about the interior noise and vibration of a metro vehicle, let alone investigations concerning a metro vehicle running on a viaduct, is noticeable. Moreover, with the trend of lighter trains and higher speeds, the increase of both air-borne and structure-borne sound will lead to the deterioration of the acoustic environment of a metro vehicle. This paper aims to determine the characteristics of the interior noise and vibration of a metro vehicle running on a viaduct. The measurements of vibration and noise were conducted in the standard B-type metro train on metro line 14 in Guangzhou (China). In order to analyze the underestimated effects of low-frequency noise components inside the vehicle, both the A-weighted SPL and linear SPL were adopted to evaluate the measured interior noise signals. The relationships between the interior noise and the vibration of the metro vehicle under various train speeds were investigated. The main noise sources of the metro vehicle were identified through the spectrum analysis of the measured vibration acceleration signals. The field measurements here can provide an available data set for researchers for further investigations, and could contribute to countermeasures for reducing interior noise and vibration of a metro vehicle.

## 2. Description of the Measurements

The main sources of interior noise can propagate by both air-borne and structure-borne paths, which includes rolling noise related to wheel and track characteristics and roughness and aerodynamic noise related to train speed and train type. The coupling effects of the train-track-bridge system may also affect the sources of interior noise when the metro vehicle is running on a viaduct [22]. The data analysis methods and the detailed measurements procedures are described here.

### 2.1. Data Analysis Methods

The total sound pressure level is a simple and direct parameter to quantify the sound level, which is defined in terms of the time-varying sound pressure level, as follows:

(1) $$SPL=10\log_{10}[\frac{1}{T}\int_{0}^{T}10^{L(t)/10}dt]$$

where T is the required period of time and L(t) is the sound pressure level at time t. This descriptor is a linear (i.e., unweighted) single value of sound level. In order to approximate the response of the human ear at low sound levels, the A-weighted total sound pressure level has been proposed and has been adopted in some international and Chinese standards. The A-weighted sound pressure level is defined as

(2) $$SPL_{A}=10\log_{10}[\frac{1}{T}\int_{0}^{T}10^{L_{PA}(t)/10}dt]$$

where $L_{PA}(t)$ is the A-weighted sound pressure level at time t according to the A-weighted circuit. 

The fast Fourier transform (FFT) is one of the most important numerical algorithms and has been widely used in signal analysis. The original signals can be transformed from the time domain to the frequency domain by adopting the FFT. Then, the spectral characteristics of the signals can be extracted. The FFT could be expressed as [37]

(3) $$X(k)=\sum_{n=0}^{N-1}x(n)W_{N}^{kn}$$

where *N* represents the order number of each harmonic component, x(n) represents the generic harmonic component as a complex number, $W_{N}^{}=e^{-j(2\pi k/N)}$ and *N* = length [x(n)]. The FFT can be used for both quantifications of a noise problem and a vibration problem. Generally, the standardized one-third-octave band is adopted in the noise spectra analysis in order to obtain more detailed information.

### 2.2. Rail Condition

**Figure 1:**
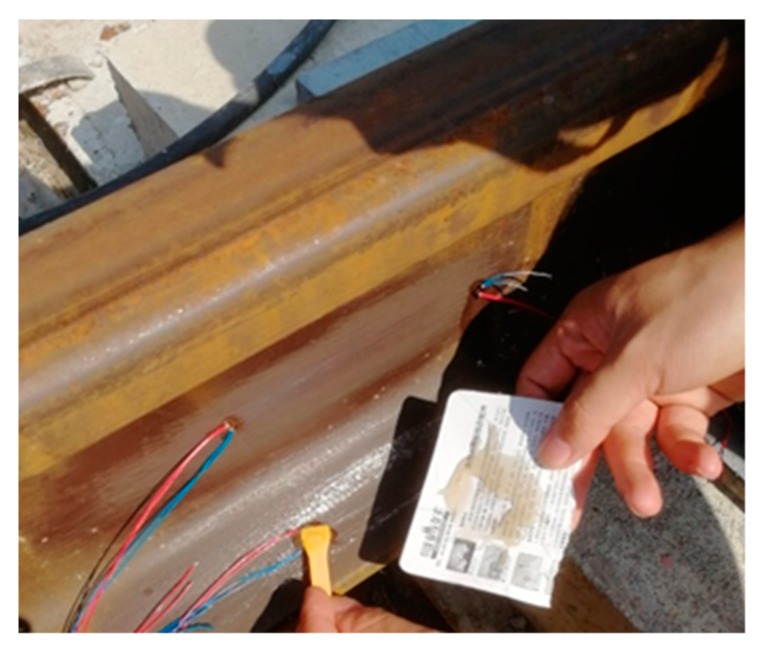
Photograph of rail condition.

Wheel–rail roughness, especially rail corrugation, is the main source of vibration excitation for the interior noise of the metro vehicle. One of the most effective and economic countermeasures to prevent rail corrugation is rail grinding, especially before the operation. The field measurement of Metro line 14 of Guangzhou was conducted two weeks before its operation, and the rails were pre-grinded before its operation, as shown in Figure 1. Therefore, the influence of rail corrugation on the internal noise and the vibration of the subway vehicles could be considered as invariant during the field measurement.

### 2.3. Continuous Rigid-Frame Box-Girder Bridge

**Figure 2:**
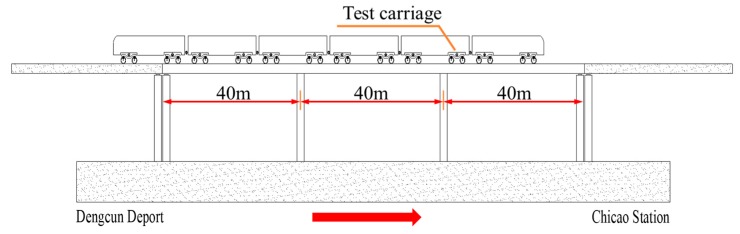
The schematic diagram of the running test.

**Figure 3:**
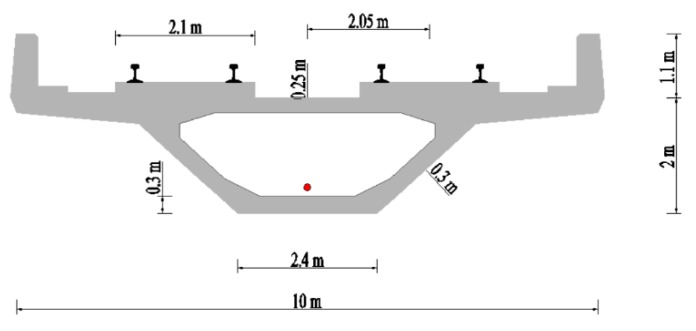
Schematic diagram of the box girder.

The running tests were conducted between overnight Dengcun station and Chicao station on metro line 14 in Guangzhou. The test section was an elevated metro line, which was mainly composed of 3 × 40 m concrete continuous rigid-frame box-girder bridge, as illustrated in Figure 2. The cross-sectional dimensions of the box girder are shown in Figure 3. The widths of the bridge deck and the bottom slab are 10 m and 2.4 m, respectively. The height of the box-girder is about 2 m. Two track systems are installed symmetrically on the bridge deck. The width of each track system is about 2.1 m. The distance between the central lines of the girder and the track system is 2.05 m. 

### 2.4. Metro Vehicle

**Figure 4:**
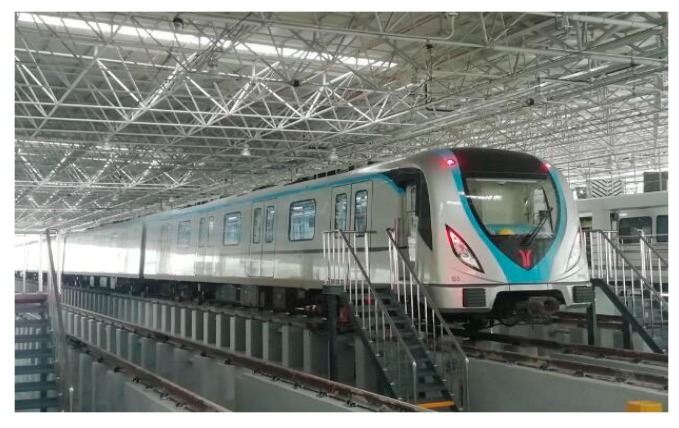
Photograph of the standard B-type metro train.

**Figure 5:**
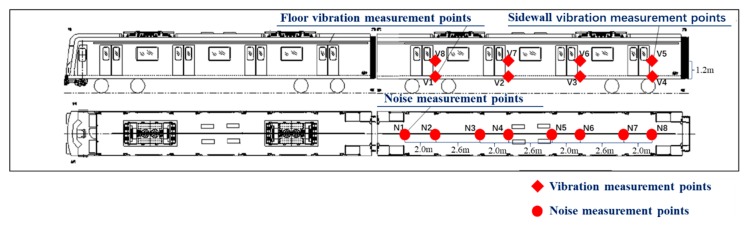
Layout of the noise and the vibration measurement points inside the vehicle.

**Figure 6:**
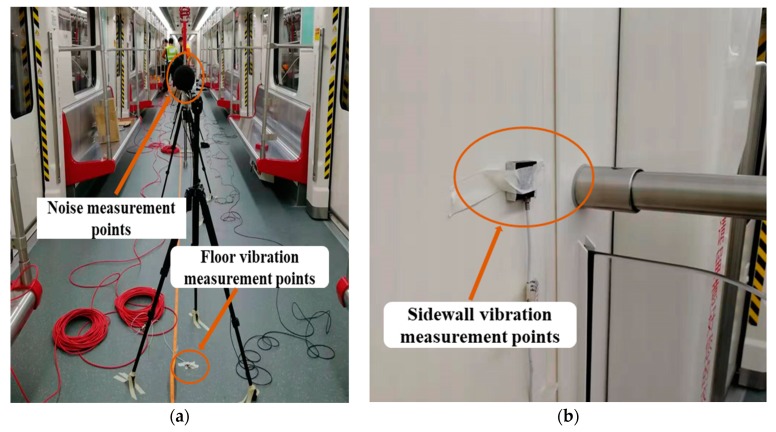
Photograph of equipment in the vehicle. (**a**) Noise measurement points and floor vibration measurement point; (**b**) sidewall vibration measurement point.

The standard B-type metro train was used in the field test, which consists of four motor vehicles and two trailer vehicles, as illustrated in Figure 4. The head and trail are trailers, and 4 motor vehicles were arranged between the two trailer cars. The width and length of each vehicle are 2.8 m and 19.98 m, respectively. The maximum speed of the B-type metro train is designed to be 120 km/h. The measurement was conducted in the second passengers’ carriage. Figure 5 and Figure 6 demonstrate the installation positions of the microphones and accelerometers in the test vehicle. Eight microphones were placed along the centerline of the metro vehicle with distance at a distance of 2 m or 2.6 m, as illustrated in Figure 5. All of these microphones were 1.2 m above the floor according to GB 14892 [31] and ISO 3381 [32]. Two microphones labeled N1 and N8 were installed at both ends of the vehicle. Two microphones labeled N2 and N7 were placed near the top of the bogie frame. The acoustic signals inside the test vehicle were collected by a 24-bit intelligent acquisition and signal processing system (type: INV3020). Four vibration measuring points (V1 to V4) were arranged on the floor directly below microphones labeled N2, N4, N6, and N8, respectively, as shown in Figure 5. There were four other vibration measuring points (V5–V8) installed at the wall panel with the same height of the microphone corresponding to four floor measuring points, respectively. The vibration signals of the metro vehicle at different speeds were measured by the SDI Model 2210 accelerometers (4 mV/g sensitivity, ±2 g full scale). The test speed was controlled to be 20 km/h, 40 km/h, 50 km/h, 60 km/h, 80 km/h, or 115 km/h. A sound level calibrator was adopted to calibrate all the microphones before and after each running test, and the deviation between the calibration before and after the tests was less than 0.5 dB. 

## 3. Results and Discussion

### 3.1. Interior Noise Spectra of the Metro Vehicle

**Figure 7:**
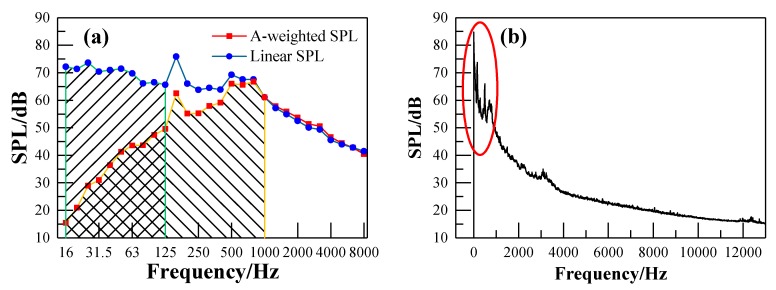
Interior noise at point N1 under the train speed of 50km/h: (**a**) one-third-octave band spectrum; (**b**) FFT spectrum.

**Figure 8:**
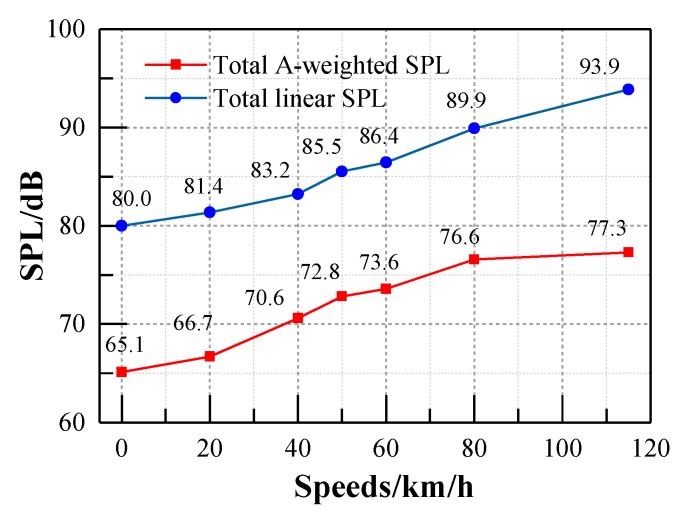
The total sound pressure levels at point N1 in respect of different train speeds.

**Figure 9:**
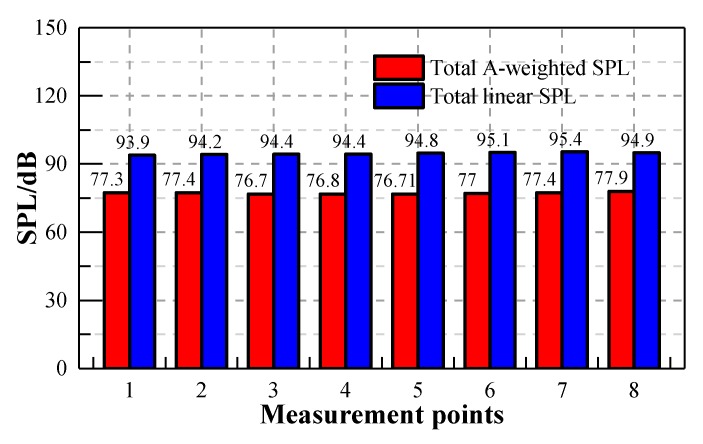
Comparison of total sound pressure levels using different methods with respect to different measuring points under the train speed of 115 km/h.

According to the Noise Limit and Measurement for Trains of Urban Rail Transit standard [31] and referring to ISO 3381 standard [32], the noise measurement procedures satisfy the requirement that background noise is 10 dB lower than the interior noise. The A-weighted SPL has been widely used to evaluate the acoustic environment inside the train, and it has already been incorporated into some international and Chinese standards due to its consistency with the auditory characteristics of humans. The A-weighted SPL underestimates the noise below 1000 Hz, while, the energy of the noise inside the metro vehicle was mainly focused on the middle and low frequency regions, as shown in Figure 7. It can be seen from Figure 8 that the phenomenon of neglect of low frequency noise in the A-weighted SPL seems to be more obvious with the increase of train speed. Figure 9 shows the total A-weighted SPL of all the measuring points and its corresponding linear SPL under the train speed of 115 km/h. The acoustic comfort inside the train may not be achieved even though the A-weighted SPL inside the train meets the requirements of these standards due to the underestimated impacts of the low-frequency component of the interior noise on people in the A-weighted SPL evaluation. Therefore, both the linear SPL and A-weighted SPL were adopted here in order to reveal the effects of A-weighted SPL’s correction in the low frequency range on the interior noise.

Since the combined effects of noise level and its frequency characteristics determines the acoustic environment of the vehicle, the total SPL and the spectrum characteristics of the interior noise are analyzed in this paper. The total SPL of each measuring point inside the vehicle with respect to different speeds is illustrated in Table 1 and Table 2. It should be noted that 0 km/h represents the interior noise of the test vehicle when it is stationary with the operation of the auxiliary equipment in the vehicle. The A-weighted SPL of the elevated metro vehicle cannot exceed the limitation of 75 dBA [31]. It can be seen from Table 2 that the A-weighted SPL meets the limit requirements of no more than 75 dBA when the vehicle speed is less than 70 km/h. When the vehicle speed exceeds 80 km/h, the A-weighted SPL does not satisfy the requirement. It indicates that countermeasures should be taken. The A-weighted SPL increases with the increasing of the train speed. Similar results of Figure 8 can also be found regarding other measuring points according to the results illustrated in Table 1 and Table 2. It can also be observed that the total SPL of all these measurement points has little difference under the same speeds. This indicates the uniform distribution of the interior noise inside the vehicle. Table 1 also show that the SPL is above 85 dB when the train speed reaches 50 km/h. Generally, long-term exposure to noise above 85 dB may damage hearing [16]. Therefore, noise reduction countermeasures are necessary during the operation of a metro line with a relatively high train speed.

**Figure 10:**
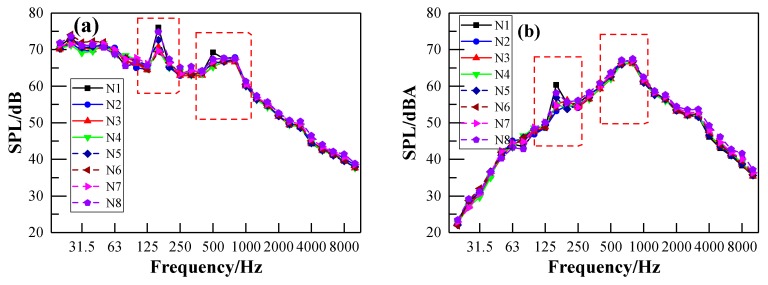
One-third-octave interior noise spectrum at different points (N1-N8) under the train speed of 50 km/h: (**a**) linear SPL; (**b**) A-weighted SPL.

**Figure 11:**
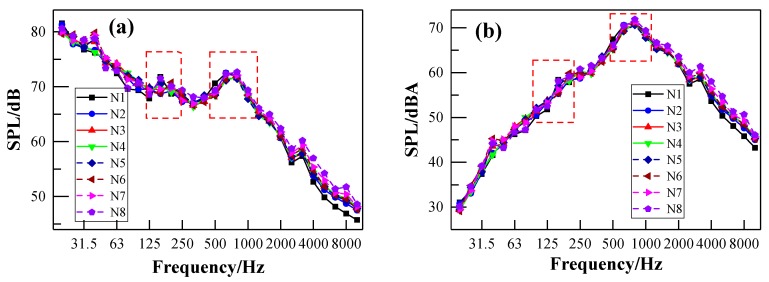
One-third-octave interior noise spectrum at different points (N1–N8) under the train speed of 115 km/h: (**a**) linear SPL; (**b**) A-weighted SPL.

Figure 10 and Figure 11 show the interior noise spectrum corresponding to the train speeds of 50 km/h and 115 km/h, respectively. It can be seen from Figure 10a and Figure 11a that the noise energy is mainly concentrated in the frequency range of 31.5–1000 Hz. There are two obvious peaks located at almost the same frequency in both interior noise spectra, as illustrated in Figure 10. A similar result can also be observed in Figure 11. However, it should be noted that the first peak in Figure 11b is not obvious. The first and second frequency peak in both Figure 10 and Figure 11 center near 160 Hz and 800 Hz, respectively. This indicates that the interior noise of the metro vehicle is mainly composed of low and medium frequency components, and the characteristics of the interior noise spectrum are not related to the train speed. The first and second frequency peaks may be generated by the vibration of the vehicle itself and wheel–rail noise, respectively. As the train speed increases, the value of the first peak gradually decreases and tends to disappear, while the value of the second peak tends to increase. The wheel–rail noise may dominate in the interior noise of the metro vehicle as the train speed increases. 

**Figure 12:**
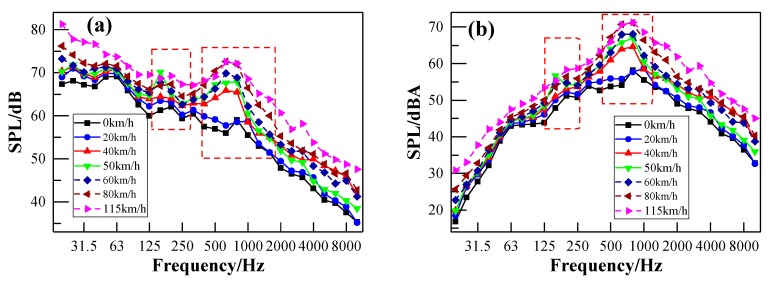
One-third-octave interior noise spectrum of point N2 under different train speeds: (**a**) linear SPL; (**b**) A-weighted SPL.

**Figure 13:**
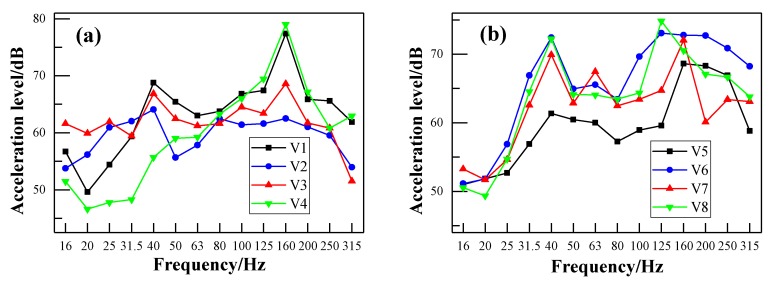
One-third-octave acceleration spectrum of different points at train speed of 50 km/h: (**a**) measuring points of the floor; (**b**) measuring points of the sidewall.

Figure 12 shows the interior noise spectrum of N2 with respect to different train speeds. It can be observed that the results of SPL are nearly independent of train speed when the train speed is less than 20 km/h, especially above 500 Hz. This indicates that the interior noise is mainly caused by the auxiliary equipment of the vehicle when the train speed is under 20 km/h. Figure 13 also illustrates the dependence of the SPL on train speed. The SPL increases with increasing train speed. There are also two frequency peaks located around 160 Hz and 800 Hz, as shown in Figure 12. The frequency peaks are nearly independent of train speed. It is worth noting that the first frequency peak appears when the train speed is 50 km/h. In the high-frequency range, the interior noise at 115 km/h is at a relatively higher level than the interior noise at other train speeds, which may be due to the increase of aerodynamic noise. However, the curve of the noise spectrum decreases rapidly above 1250 Hz at these speeds due to the good sound insulation of the vehicle itself for high-frequency noise. 

### 3.2. Vibration Spectra of the Metro Vehicle

**Figure 14:**
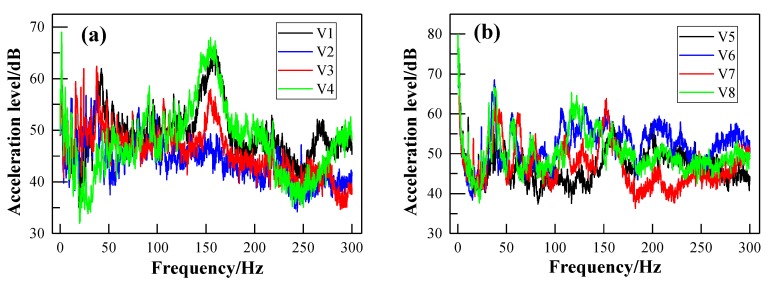
Fast Fourier transform (FFT) spectra of different points at train speed of 50 km/h: (**a**) measuring points of the floor; (**b**) measuring points of the sidewall.

The propagation path of noise in trains can be generally divided into the structural sound transmission and air sound transmission. The aim of vehicle vibration measurements is to reveal the relationship between the interior noise and the vibration of the metro vehicle. Figure 13a,b shows the vibration levels of the floor and sidewall, respectively, at a train speed of 50 km/h. Their corresponding frequency spectra using the FFT method are illustrated in Figure 14a,b, respectively. It can be observed that the vibration energy of the floor is mainly concentrated in the frequency range of 100–200 Hz. The frequency peak is located at 160 Hz, which corresponds to the first frequency peak of the interior noise spectrum in Figure 10. This means that the low-frequency noise may be generated by the vibration of the floor. Figure 13a and Figure 14a show that the vibration peak of the measuring points nearing the bogie frame (V1 and V4) are higher than the measuring points in the middle of the vehicle (V2 and V3). This means that the vibration of the bogie frame caused by the wheel–rail excitation may be directly transmitted to the vehicle and stimulate the vibration of the floor. It can be seen in Figure 14a that the sidewall has one more obvious frequency peak around 40 Hz, apart from the same frequency peak as the floor. This kind of situation may be due to the secondary vibration of the sidewall caused by air sound. However, there is no significant difference in the vibration level of each measuring point around 40 Hz after applying the FFT transformation, as shown in Figure 14b. This may be the relatively low air-borne sound energy compared with the vibration energy of the bogie frame. 

**Figure 15:**
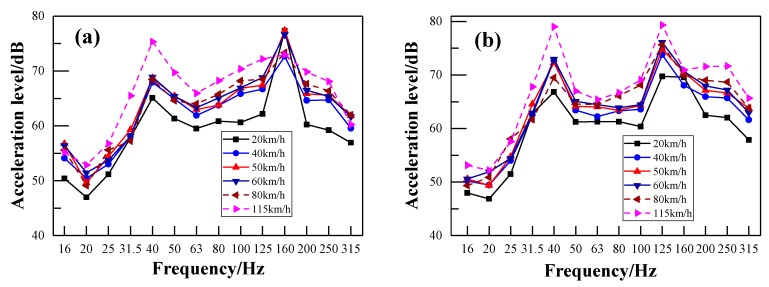
One-third-octave acceleration spectrum in respect of different train speeds: (**a**) measuring point V1 of the floor; (**b**) measuring point V8 of the sidewall.

**Figure 16:**
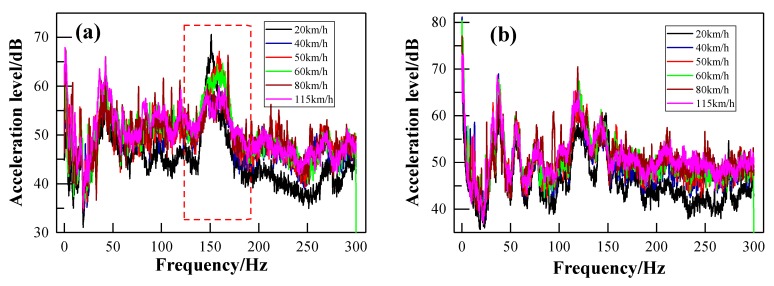
FFT spectra in respect of different train speeds: (**a**) measuring point V1 of the floor; (**b**) measuring point V8 of the sidewall.

Figure 15a,b shows the acceleration level of the floor measurement point V1 and the sidewall measurement point V8, respectively, with respect to different train speeds. Their corresponding frequency spectra using the FFT method are illustrated in Figure 16a,b, respectively. It can be seen from Figure 15 that both frequency peaks (around 40 Hz and 160 Hz) of the floor and sidewall are independent of the train speed. However, the vibration acceleration level increases with the increase of the train speed in both the low-frequency and high- frequency range. However, the vibration acceleration level increases with the increase of the train speed in both the low-frequency range and the high- frequency range. The acceleration level difference between the train speeds of 40 km/h and 80 km/h is not obvious. However, the acceleration level is much higher when the train speed reaches 115 km/h. This may be the instability of the vehicle itself at a relatively higher speed. Figure 16a shows that the acceleration levels of the floor around the second frequency peak decrease with the increase of the train speed, which may be the rolling effects of the rail. It can be seen from Figure 16b that the acceleration levels of the sidewall are nearly the same below 80 Hz. Furthermore, the acceleration levels between train speeds are also not obviously different above 80 Hz, except at 20 km/h. This indicates that the characteristics of the vehicle itself dominate the acceleration levels of the sidewall. 

## 4. Conclusions

Field experiments of the interior noise and vibration of a standard B-type metro train running on a viaduct were undertaken overnight on the Metro line 14 of Guangzhou (China). Both the A-weighted SPL and linear SPL were adopted to evaluate the measured interior noise signals. The FFT method was applied to measure vibrations of both the vehicle’s floor and sidewall. The results show that the interior noise increases sharply with the increasing train running speed. However, the effects of the train’s running speeds on the acceleration levels of the floor and sidewall are not apparent, especially in the range of 40–80 km/h. There are two obvious ranges (125–250 Hz and 400–1000 Hz) in the frequency domain of the interior noise. Their corresponding center frequencies are 160 Hz and 800 Hz, respectively. These two frequency peaks are nearly independent of train speed. The spectrum analysis of the vehicle body vibration shows that the frequency peak of the floor corresponds to the first frequency peak of the interior noise spectrum. This indicates that the vibration of the floor contributes to the low-frequency noise components of the interior noise. There are two frequency peaks of the sidewall’s acceleration level, around 40 Hz and 160 Hz. The frequency peaks of the floor and sidewall are also independent of the train speed. This indicates that the characteristics of the vehicle itself dominate the frequency peaks of the acceleration levels of the floor and sidewall. The results show that different vibration reduction measures should be taken according to the characteristics of the floor and sidewall. Since field measurements of the interior noise and vibration of metro vehicles are quite rare in the literature, this paper can also provide an available data set and reference for researchers in further investigations.

## Figures and Tables

**Table 1:** Total linear sound pressure level (SPL) of each measuring point with respect to different train speeds (dB).

Points	Speeds						
	0 km/h	20 km/h	40 km/h	50 km/h	60 km/h	80 km/h	115 km/h
N1	80.0	81.4	83.2	85.5	86.4	89.9	93.9
N2	79.5	80.6	82.9	85.1	86.2	89.8	94.2
N3	79.5	80.5	82.7	84.9	86.1	89.6	94.4
N4	79.4	80.5	82.7	84.9	86.1	89.5	94.4
N5	79.3	81.0	83.7	85.5	86.7	89.9	94.8
N6	80.0	81.3	83.7	85.8	86.9	90.2	95.1
N7	79.9	81.3	83.7	85.9	87.0	90.5	95.4
N8	79.1	81.0	83.9	85.7	86.7	90.1	94.9

**Table 2:** Total A-weighted SPL of each measuring point with respect to different train speeds (dBA).

Points	Speeds						
	0 km/h	20 km/h	40 km/h	50 km/h	60 km/h	80 km/h	115 km/h
N1	65.1	66.7	70.6	72.8	73.6	76.6	77.3
N2	65.1	66.2	70.6	72.6	73.4	76.4	77.4
N3	64.8	66.2	70.0	72.1	72.8	75.7	76.7
N4	64.8	66.4	70.3	72.2	72.9	76.0	76.8
N5	65.0	66.8	70.3	72.4	73.0	76.0	76.7
N6	64.9	66.9	70.3	72.5	72.9	76.1	77.0
N7	64.4	67.1	70.6	72.8	73.4	76.3	77.4
N8	64.0	67.8	70.8	73.3	73.9	76.9	77.9
